# Improvement analysis of organic light emitting diode temperature control by integrating whale algorithm in PID control system

**DOI:** 10.1371/journal.pone.0327851

**Published:** 2025-07-22

**Authors:** Dayu Zhang, Cong Guan

**Affiliations:** 1 School of Intelligent Manufacturing and Elevator, Huzhou Vocational and Technical College, Huzhou, China; 2 Faculty of Robot Science and Engineering, Northeastern University, Shenyang, China; National Institute of Technology Warangal, INDIA

## Abstract

Organic Light-Emitting Diode (OLED) is a high-performance display technology. Its performance and lifespan are extremely sensitive to the operating temperature. The existing temperature control methods, such as the traditional Proportional-Integral-Derivative (PID) controller, are difficult to meet the requirements of OLED for precise temperature control, especially in systems with significant nonlinear and time-varying characteristics. To solve this problem, the study proposes an improved PID controller based on the Long Short-Term Memory (LSTM) optimized by the Whale Optimization Algorithm (WOA). This method combines the global optimization ability of WOA and the timing analysis ability of LSTM. By optimizing the parameters of the PID controller, the accuracy and adaptability of temperature control are improved. Meanwhile, the effectiveness of the proposed controller is verified by constructing a thermodynamic model and combining experimental data. In the experimental results, compared with the traditional PID controller, the overshoot of the WOA-LSTM-PID controller was reduced from 8.5°C to 0.3°C, the steady-state error was reduced from 1.2°C to 0.2°C, the regulation time was shortened from 42.5 seconds to 20.2 seconds, and the response time was shortened from 70.5 seconds to 21.9 seconds. Furthermore, the root mean square error has been reduced from 5.23°C of the traditional PID to 0.78°C. The research results show that the WOA-LSTM-PID controller can significantly improve the accuracy and stability of OLED temperature control, while reducing the regulation time and response time. This controller effectively addresses the nonlinear and time-varying characteristics in OLED temperature control by optimizing the PID parameters. The innovation of the research lies in the combination of the WOA and the LSTM network. By optimizing the parameters of the PID controller, high-precision control of the OLED temperature has been achieved. This study not only proposes a new theoretical optimization method but also verifies its significant performance improvement in experiments. Furthermore, this method has strong universality and can be applied to other temperature-sensitive systems.

## 1. Introduction

The modern display technology has made Organic Light-Emitting Diodes (OLEDs) a new star in display technology, since their unique advantages, including self-emission, high contrast, low power consumption, fast response time, and thin design. The development of OLED display technology not only drives innovation in smartphones, televisions, and wearable devices but also plays an important role in future flexible and transparent display technologies [1,2]. However, the performance and lifespan of OLEDs are significantly affected by the operating temperature. Even small fluctuations in temperature can reduce device performance and even damage, making temperature control a key technology in OLED applications [3,4]. Despite the rapid development of OLED technology, its temperature control technology is not yet fully mature. The existing temperature control methods, such as traditional Proportional-Integral-Derivative (PID) control, often struggle to meet the strict requirements of OLED for precise temperature control [5,6]. Although PID controllers are widely used in industry, their parameter adjustment relies on experience and trial and error, making it difficult to cope with the nonlinear and time-varying characteristics in OLED temperature control. In addition, with the diversification and complexity of OLED applications, traditional temperature control methods are facing more severe challenges in terms of real-time, accuracy, and adaptability [7,8].

At present, scholars have researched the temperature control of OLEDs. Chen J et al. proposed a method based on alternating positive and negative pulse voltage supply to achieve efficient OLEDs with organic fluorescent groups. The negative pulse voltage applied could help these trapped electrons and holes with ultra-long lifetimes to escape and then recombine with each other to produce enhanced luminescence. This method captured the dynamic behavior of charges, paving the way for high-performance OLEDs operating in low-temperature environments such as outer space or aerospace [9]. Tam B-ST et al. used near-space sublimation technology as a basis to fully characterize the sublimation behavior of organic thin films under near-space sublimation based on OLED materials. Near space sublimation technology had the characteristics of low temperature, high material utilization rate, and short process time, which could become a display material for manufacturing flexible OLEDs [10]. Hillebrandt et al. proposed that plasma treatment and the implementation of a silver intermediate layer cause ohmic contact conditions. This method could promote direct vacuum deposition of orange and blue emitting OLED stacks, thereby achieving micrometer-level pixels on the chip, while also becoming more precise in temperature control [11]. Wang Q proposed an optimized PID precision lubrication controller to lower the impact of the environment on the flow rate of lubricating oil. The system simulated the flow dynamics of biodegradable lubricants with temperature changes and proposed an improved PID algorithm that utilized speed feedback to improve the tracking and interference suppression capabilities of the set point. Compared with traditional PID methods, the flow control accuracy of this method has been improved by 0.8%. The on-site testing conducted within the fluctuation interval of −3 ~ 35°C verified the robustness of this method in keeping a constant droplet velocity [12]. Wang Y et al. constructed a heating process model for OLED glass substrates built on a heat pipe array to investigate the thermal characteristics, as the temperature uniformity during heat treatment can affect imaging display quality. This model could reduce the heating temperature distinction of the glass substrate to an optimal level [13]. ChaturvediS et al. proposed a dual-feedback PID controller model based on the teaching-learning optimization algorithm in view of the current situation of PID controller parameter tuning in nonlinear ball and beam systems. This model included two PID controllers, which were respectively used to control the position of the ball and the Angle of the motor. The parameters of the PID controller were optimized through the teaching-learning optimization algorithm to achieve a fast and stable system response. The results showed that the PID controller optimized by the teaching learning optimization algorithm outperformed the PID controller optimized by the traditional Zeigler Nichols method and Particle Swarm Optimization (PSO) algorithm in performance indicators such as rise time, overshoot time, and adjustment time. Moreover, the optimized controller exhibited good robustness in the presence of disturbances and parameter uncertainties [14]. Chaturvedi S et al. artificially proposed a novel PID Neural Network model based on PSO (PSO-PID-NN) in view of the current situation of temperature control in the jacket-type Continuous Stirred Reactor (CSTR). This model included a simple neural network structure, containing only three hidden layer neurons and one output neuron. This method utilized PSO to optimize the weights of the output layer to adjust the PID gain, and adopted the Mean Square Error (MSE) as the objective function to optimize the weights. The results showed that, compared with the traditional Zeigler Nichols-tuned PID controller and the NN-PID controller based on Backpropagation (BP-NN-PID), the overshot of the proposed PSO-PID-NN controller was 23.13%, while that of the BP-NN-PID was 26.33%. Zeigler Nichols tuned the PID controller to 44.13%. Furthermore, the rise time of PSO-PID-NN was 0.1283 seconds, while that of BP-NN-PID was 0.2727 seconds, and that of the Zeigler Nichols-tuned PID controller was 0.2813 seconds. In the anti-interference ability test, the PSO-PID-NN controller also demonstrated higher efficiency than the BP-NN-PID and Zeigler Nichols-tuned PID controllers [15].

The above research has achieved certain results in OLED temperature control, but the current existing temperature control methods, such as traditional PID control, are often difficult to meet the strict requirements of OLED for precise temperature control. Meanwhile, traditional PID temperature controllers have weak control capabilities for nonlinear systems. In some complex nonlinear systems, traditional PID temperature controllers may not be able to provide stable temperature control. Although PID controllers are widely used in industry, their parameter adjustment relies on experience and trial and error, making it difficult to cope with the nonlinear and time-varying characteristics in OLED temperature control [16]. In addition, with the diversification and complexity of OLED applications, traditional temperature control methods are facing more severe challenges in terms of real-time, accuracy, and adaptability. Based on this, this study proposes an improved PID controller for optimizing Long Short-Term Memory (LSTM) using Whale Optimization Algorithm (WOA). The advantage of this algorithm lies in combining the global optimization ability of WOA and also taking advantage of the powerful processing ability of LSTM for time series data, thereby achieving innovation in the control strategy. Meanwhile, the learning rate and the number of neuron layers of the LSTM network are optimized through WOA, further improving the performance of the model. This intelligent optimization process enables the PID controller to better adapt to complex nonlinear systems. The contributions of this study are:(1) An improved PID controller (WOA-LSTM-PID) based on the whale algorithm optimization of long and short-term memory network is proposed, which introduces a new control strategy for the field of temperature control of organic light-emitting diodes (OLEDs), and enriches the system of control methods in this field. (2) Compared with existing studies, the WOA-LSTM-PID controller performs well in terms of temperature control accuracy and stability, and is able to effectively cope with the nonlinear and time-varying characteristics in OLED temperature control. (3) The thermodynamic model constructed in this study has high accuracy and can accurately describe the working process of the OLED temperature control system. This study aims to develop a novel temperature control strategy to lift the temperature control stability of OLEDs. By introducing advanced control algorithms and optimization techniques, OLED provides a more precise, responsive, and adaptable temperature control solution. The practical significance of the research lies in that by achieving precise temperature control, manufacturers can enhance product consistency, reduce production costs, and extend the service life of OLED devices.

## 2. Methods and materials

### 2.1 Design of OLED temperature control system

**Fig 1:**
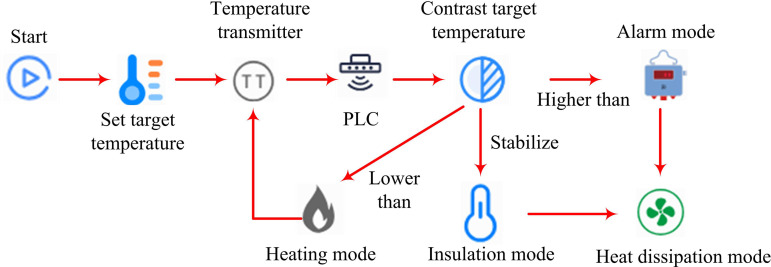
Workflow of OLED temperature control system.

In the temperature control experiment of OLED, small fluctuations in temperature can affect the device performance of light-emitting diodes. Usually, when the experimental temperature exceeds the limit temperature of OLED devices by 1°C, the device is damaged and its photoelectric performance undergoes a sudden change [17,18]. This study designs a temperature control system based on OLED technology. The system mainly consists of heating mode, insulation mode, cooling mode, and alarm module. The workflow of the OLED temperature control system is shown in Fig 1.

In Fig 1, the heating mode is mainly based on an electrically heated aluminum block. By issuing a heating command, the temperature reaches the set value, and the temperature inside the device is displayed in real-time in the operation console of the backend staff [19,20]. The insulation program mainly detects that the temperature has reached the set value and adjusts the power of the heating module to limit the temperature from continuing to rise. The cooling mode achieves device cooling by opening the pipeline and connecting nitrogen gas. Finally, in the alarm module, when the temperature exceeds the maximum value, the temperature control system emits light and sound alarms, and the heat dissipation mode is turned on to control the temperature and pressure [21,22]. In the experimental setup, all thermal energy comes from the electric heating block. Based on relevant thermodynamic laws, the heat generated by this module is composed of both the heat loss during heating and the heat generated within the device. The relevant mathematical expression is given by formula (1).


$$\left\{ \begin{array}{c} Q_{Heat}=Q_{loss}+Q_{In}\\ Q_{loss}=Q_{con}+Q_{rad} \end{array}\right.$$ (1)

In formula (1), $Q_{Heat}$ is the heat of the heating block. $Q_{Heat}$ is heat loss. $Q_{In}$ is the heat of the internal air. $Q_{con}$ is the heat generated by thermal convection. $Q_{rad}$ is the heat of thermal radiation. Among them, the mathematical expression of heat based on thermal convection is shown in formula (2).


$$Q_{con}=oS(T-T_{0})t$$ (2)

In formula (2), o is the heat transfer coefficient. S is the heat dissipation area. T and $T_{0}$ are the temperature rise and initial temperature. t is the temperature rise time k. Combining the heat transfer coefficient and thermal conductivity, the mathematical expression of heat convection can be optimized as formula (3).


$$\begin{array}{c} Q_{con}=1/\left(\frac{d_{2}}{o_{1}d_{1}}+\frac{d_{2}}{2\lambda }In\left(\frac{d_{2}}{d_{1}}\right)+\frac{1}{o_{2}}\right)(2_{\pi }^{d22}H+2\pi d_{2}H)(T-T_{0})t \end{array}$$ (3)

In formula (3), $d_{1}$ and $d_{2}$ are the inner and outer diameters of the experimental chamber. $o_{1}$ and $o_{2}$ are the natural convection coefficients of the inner and outer air. H is the height of the temperature control device. The heat of thermal radiation obtained based on thermodynamic laws is shown in formula (4).


$$\left\{ \begin{array}{c} Q_{rad}=\varepsilon \delta S(T^{4}-T_{0}^{4})\\ \delta =5.67\times 10^{-8}w/(m^{2}\cdot h) \end{array}\right.$$ (4)

In formula (4), ε is the surface emissivity, ε=0.5. δ is the Stepan Boltzmann constant. Finally, based on formulas (3) and (4), the thermodynamic model of OLED can be obtained, as shown in formula (5).


$$\begin{array}{c} Model=1/(\frac{d_{2}}{o_{1}d_{1}}+\frac{d_{2}}{2\lambda }In(\frac{d_{2}}{d_{1}})+\frac{1}{o_{2}})(2\pi d_{2}^{2}H+2\pi d_{2}H)(T-T_{0})t\\ +\varepsilon \delta S(T^{4}-T_{0}^{4})+cm(T-T_{0}) \end{array}$$ (5)

In formula (5), Model is the thermodynamic model of OLED. λ represents the thermal diffusivity, S represents the intensity of the heat source, and c represents the specific heat capacity, which is used to describe the material’s ability to store heat. Finally, based on the obtained thermodynamic model, the stability of the constructed OLED-based temperature control system can be verified by comparing it with the actual measured temperature.

### 2.2 Determination of parameters and influencing factors based on PID temperature control system

The above study constructs a thermodynamic model for OLED. Based on this, this study defines the temperature control system as an inertial hysteresis system, so the transfer function based on the temperature control system is shown in formula (6).


$$F(s)=Ge^{\left(-\tau s\right)}/(Rs+1)$$ (6)

In formula (6), F is the transfer function, G is the amplification factor, and τ is the lag time. Based on formula (5), this study introduces the Cohen Kuhn formula to characterize the relevant parameters of the OLED temperature control system, as shown in formula (7).


$$\left\{ \begin{array}{c} G=\frac{\Delta D}{\Delta N}\\ R=1.5(t_{up}-t_{down})\\ \tau =0.5(3t_{down}-t_{up}) \end{array}\right.$$ (7)

**Fig 2:**
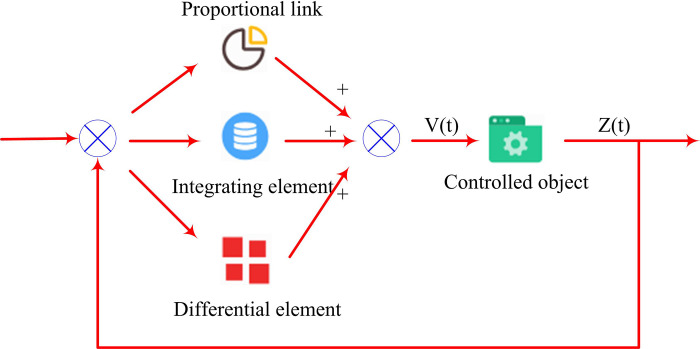
The principle of traditional PID control system.

In formula (7), ΔD denotes the original temperature of the system and the degree of change when it reaches a stable temperature. ΔD is the voltage signal of the system. $t_{up}$ is the time required for the response curve output by the temperature control system to reach the corresponding superscript value. $t_{down}$ is the time required for the corresponding curve output by the system to reach the corresponding index value. In the OLED temperature control system, this study uses a PID control system for temperature regulation, which characterizes the output of the controller through proportional, integral, and derivative forms [23,24]. The principle based on traditional PID control system is shown in Fig 2.

In Fig 2, the PID controller is used to dynamically detect the temperature control system, which can make the actual output value closer to the expected output value. The expression based on the effects of integration, differentiation, and proportionality is shown in formula (8).


$$\left\{ \begin{array}{c} V(t)=K_{p}e(t)+K_{i}\int e(t)dt+K_{d}\frac{de(t)}{dt}\\ e(t)=SP-PV \end{array}\right.$$ (8)

In formula (8), V(t) is the output of the controller, that is, the control quantity. e(t) is the error signal. SP is the set value. PV is the measured value. $K_{p}$ means the proportional gain. $K_{i}$ is the integral gain. $K_{d}$ denotes the differential gain. In practical applications, the formula of PID controller may also include some additional terms, such as integral limiting and differential feedforward, to improve the performance of the controller [25,26]. The mathematical expression of the PID controller is shown in equation (9).


$$V(n)=K_{p}e(n)+K_{i}\sum_{i=0}^{n}e(i)\Delta t+K_{d}\frac{e(n)-e(n-1)}{\Delta t}$$ (9)

In formula (9), Δt is the sampling period. The model in the study is realized in the discrete domain, which can more accurately reflect the working process of the actual control system, facilitate the implementation and debugging on the actual hardware platform, and also help to improve the real-time and accuracy of the control system. Regarding the components of a PID controller, firstly, proportion is the most direct and fundamental part of the PID controller. It adjusts the control quantity based on the current error signal e(t). The $K_{p}$ is a positive constant that determines the strength of the controller’s response to errors. Its advantage is that it can quickly respond to error signals and provide a fast initial response, but using proportional control alone often cannot eliminate Steady-State Errors (S-SE). The function of integral control is to accumulate past errors and adjust the control quantity based on the accumulated errors. The $K_{i}$ is also a positive constant, which determines the influence of the integral term on the control variable. Integral control can eliminate S-SEs, but it can lead to slower system response. Finally, differential control can predict the future trend of errors and adjust the control variables based on this trend [27,28]. The $K_{d}$ determines the influence of the differential term on the control variable, which can improve the stability and response velocity of the system, but differential control is very sensitive to noise [29]. This study introduces the Ziegler Nichols closed loop method for parameter tuning in PID controllers. Firstly, based on formula (10), the PID controller is set to pure proportional control.


$$\left\{ \begin{array}{c} K_{i}=0\\ K_{d}=0 \end{array}\right.$$ (10)

Based on formula (10), first, the $K_{i}$ and $K_{d}$ are set to 0, and then the $K_{p}$ is gradually increased until the system produces sustained oscillations under step inputs. Subsequently, the period of the system during continuous oscillation is recorded. Finally, the parameters of the PID are calculated based on the ultimate gain and oscillation period, as shown in formula (11).


$$\left\{ \begin{array}{c} K_{p}=1.20K_{u}\\ K_{i}=\frac{0.5K_{u}}{T_{u}}\\ K_{d}=0.075*K_{u}*T_{u} \end{array}\right.$$ (11)

The Ziegler Nichols method can quickly tune PID parameters without the need for complex mathematical modeling of the system. However, this method relies on the dynamic characteristics of the system, and if the system characteristics change, the PID parameters may need to be recalibrated. In terms of parameter selection, the study mainly considers the practical application requirements, technical limitations, and the applicability of the theoretical model. Among the practical application requirements, temperature control is crucial for maintaining the performance of the equipment and extending its service life. The parameter range selected for the research reflects the environmental conditions that these devices may encounter during actual operation. Secondly, different materials have different thermal conductivities, electrical conductivities, and thermal diffusivity. The parameter range selected for the study covers the typical values of these material properties to ensure the applicability of the model. Due to technological limitations, the accuracy of actual measuring equipment limits the range of parameters that can be accurately measured. Therefore, the selected parameter range ensures the reliability and stability of the measurement data. Meanwhile, the capabilities of experimental equipment and control systems also limit the range of parameters that can be processed. Finally, to make the model easy to understand and calculate, some parameters are simplified in the study to achieve the purpose of simplifying the model.

### 2.3 Improvement of OLED temperature control based on WOA

**Fig 3:**
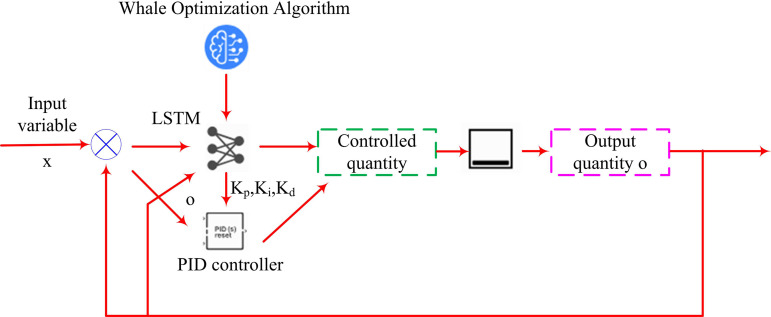
The complete process of optimizing PID control algorithm based on WOA-LSTM.

The above research discusses the shortcomings of traditional PID temperature control. This study improves the traditional PID control system by introducing WOA-LSTM to enhance the PID temperature control algorithm. Therefore, the complete process of optimizing the traditional PID control algorithm based on WOA-LSTM is shown in Fig 3.

In Fig 3, unlike traditional PID control systems, WOA-LSTM optimizes proportional gain, integral gain, and differential gain. Traditional Recurrent Neural Networks (RNNs) suffer from gradient vanishing or exploding problems when processing long sequence data, while LSTMs only need to rely on temperature data from actual processes. This study first introduces LSTM to optimize PID, which includes Input Gate (IG), Forget Gate (FG), and Output Gate (OG). Firstly, the network searches for lost information based on the FG, as shown in formula (12).


$$f_{t}=\sigma (W_{f}\cdot [h_{t-1},x_{t}]+b_{f})$$ (12)

In formula (12), $f_{t}$ is the output of the FG. σ means the sigmoid function. $W_{f}$ is the weight matrix of the FG. $h_{t-1}$ denotes the hidden state of the previous time step. $x_{t}$ is the input for the current time step. $b_{f}$ is the bias of the FG. When the information transitions to the IG, its output is shown in formula (13).


$$i_{t}=\sigma (W_{i}\times [h_{t-1},x_{t}]+b_{i})$$ (13)

In formula (13), $i_{t}$ is the output of the IG. $W_{i}$ and $b_{i}$ are the IG’s weight matrix and bias. In addition, there is a new candidate value involved in the IG, as shown in formula (14).


$$C_{t}^{\sim }=\tanh(W_{C}\cdot [h_{t-1},x_{t}]+b_{C})$$ (14)

In formula (14), $C_{t}^{\sim }$ is the candidate cell state. tanh is the activation function. $W_{C}$ is the $C_{t}^{\sim }$ ’s weight matrix. $b_{C}$ refers to the bias of $C_{t}^{\sim }$. Subsequently, the unit state based on the current time step is shown in formula (15).


$$C_{t}=f_{t}\cdot C_{t-1}+i_{t}\cdot C_{t}^{\sim }$$ (15)

In formula (15), $C_{t}$ and $C_{t-1}$ are the unit states of the current and previous time steps. Finally, by combining the input data with the unit state, the output value of the OG can be obtained, as give by formula (16).


$$\left\{ \begin{array}{c} o_{t}=\sigma (W_{o}\cdot [h_{t-1},x_{t}]+b_{o})\\ h_{t-1}=o_{t}\cdot \tanh(C_{t}) \end{array}\right.$$ (16)

**Fig 4:**
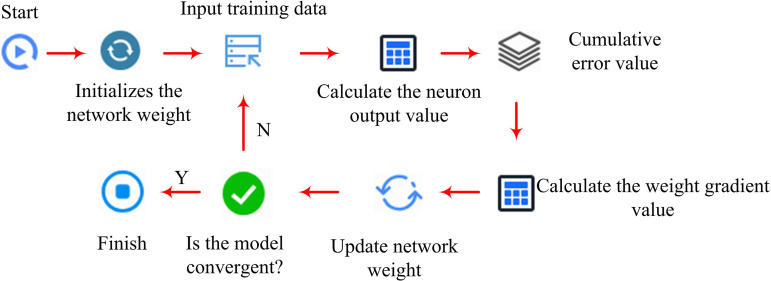
LSTM-optimized PID controller flow.

In formula (16), $o_{t}$ and $W_{o}$ are the output and weight matrix of the OG. $b_{o}$ is the reset of the OG. $h_{t}$ means the hidden state of the current time step. Therefore, the process of optimizing the PID controller based on LSTM is shown in Fig 4.

**Fig 5:**
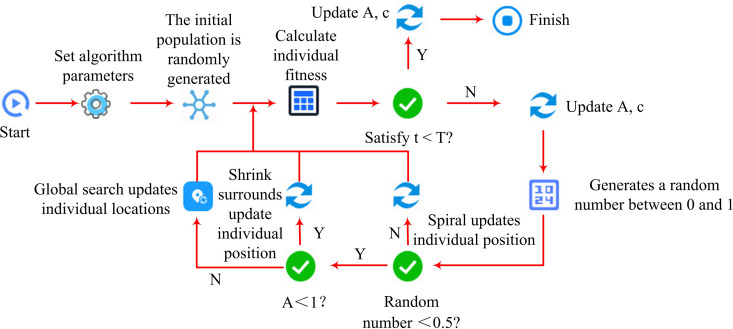
Process based on WOA-LSTM-PID model.

Fig 4 shows the training flowchart of LSTM. The core idea of temperature control based on LSTM optimized PID is to improve the accuracy and robustness. Firstly, in terms of robustness, LSTM can learn from historical data and improve the system’s robustness by handling unknown factors. Secondly, in terms of adaptive control, LSTM can maintain the system in a stable state by adjusting parameters. Finally, the neural network can predict the temperature changes of OLED, allowing the PID controller to make corresponding adjustments based on the predicted data of the neural network. This paper aims to improve the predictive performance of neural networks for temperature by introducing WOA to perfect the learning rate and number of neuron layers of LSTM. This algorithm seeks the optimal solution by mimicking the hunting behavior of humpback whales in the ocean, including the search, encirclement, and attack processes of whale populations. WOA has the advantages of simple operation, fewer parameters that need to be set, and strong optimization ability. The flow of the WOA-LSTM-PID model is displayed in Fig 5.

**Fig 6:**
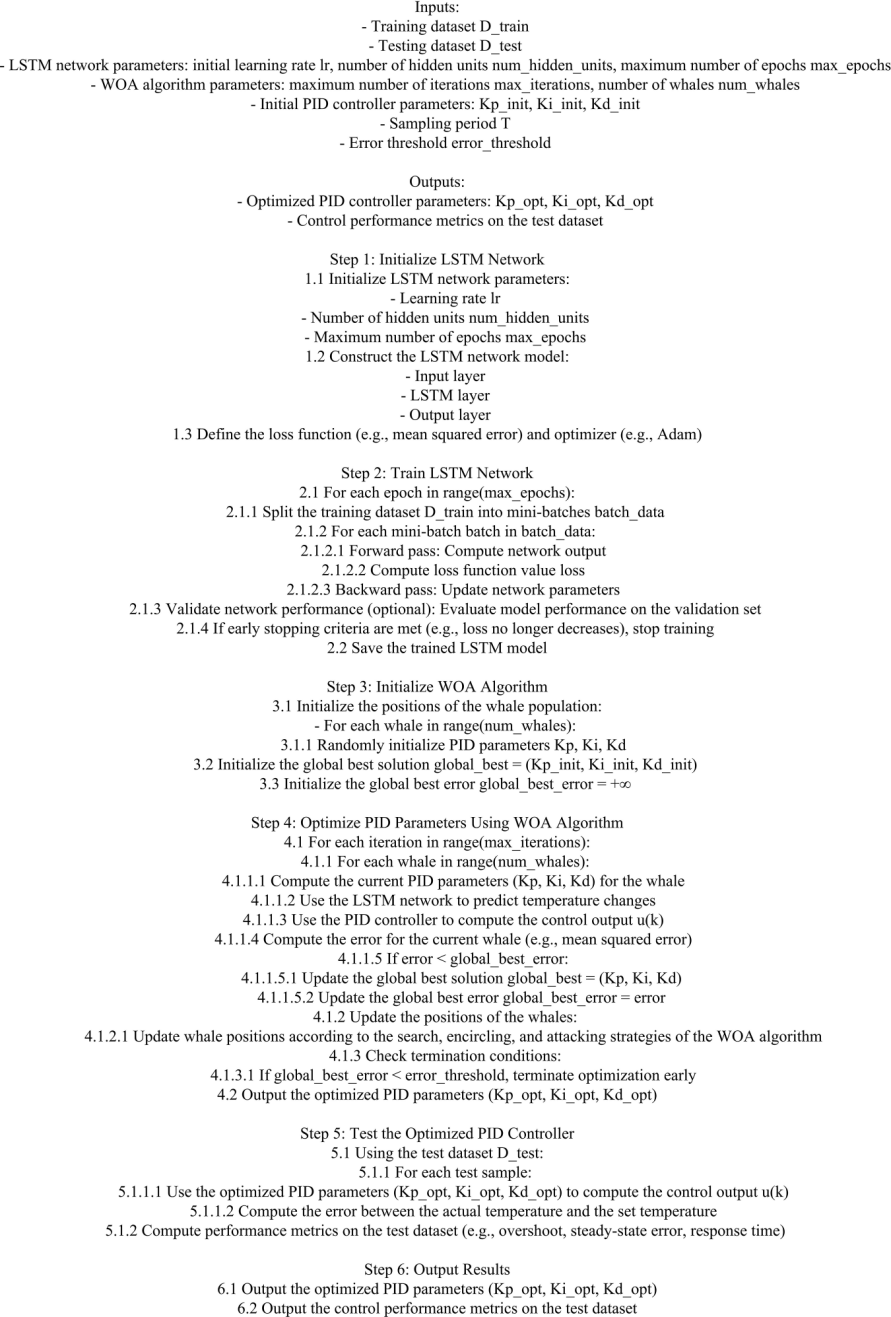
The pseudo-code of the WOA-LSTM-PID model.

In Fig 5, the process of improving the PID based on WOA-LSTM first clarifies the error function as the optimization objective. Secondly, the learning rate and number of neuron layers of the neural network need to have a clear search space. Subsequently, after initializing the parameters, iterative optimization is carried out based on WOA, followed by determining the termination conditions. Stopping iterating when the error function reaches a small value or the iteration reaches a certain value. After the iteration is completed, the whale with the best fitness is defined as the optimal solution, and the LSTM is updated with the parameters corresponding to the optimal solution to complete the optimization of the temperature control system under PID. Finally, the neural network optimized by WOA is tested to evaluate its performance optimization. Finally, the pseudo-code based on the WOA-LSTM-PID model is shown in Fig 6.

## 3. Results

### 3.1 Feasibility verification of thermodynamic model

**Fig 7:**
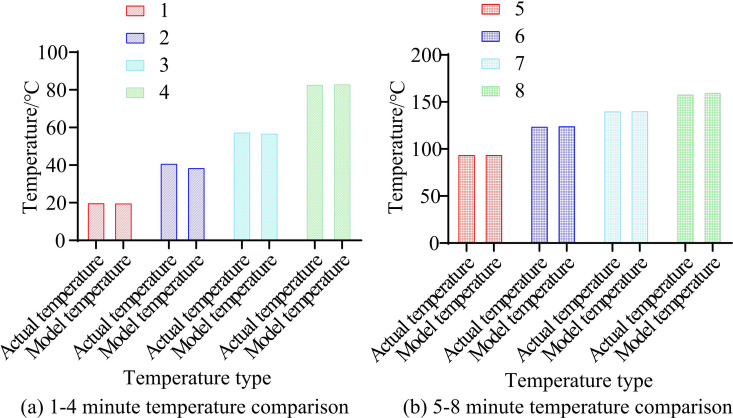
1–8 minute temperature comparison.

This study first verifies the feasibility of the model by measuring the actual temperature inside the cavity for 1–8 minutes and observing the difference between the two to determine the feasibility of the research model, as shown in Fig 7.

Fig 7(a) and 7(b) show the comparison between actual temperature and model temperature within 1–4 minutes and 5–8 minutes. In Fig 7(a), during the initial heating stage, the model temperature is lower than the actual temperature. This may be due to the fact that as the temperature increases, the heat transfer coefficient and convective heat transfer coefficient increase, resulting in the model temperature being lower than the actual measured temperature. In the initial stage of temperature rise, the thermal conductivity and convective heat transfer coefficient may be lower than expected. This may be because at low temperatures, the thermal conductivity of the material and the convective heat transfer capacity of the air may not have reached the optimal state. Meanwhile, the thermal inertia of the system may cause the temperature response to lag behind the model prediction. Greater thermal inertia signifies that the system requires a greater temporal span to absorb and distribute heat, resulting in discrepancies between model predictions and actual measurements. In Fig 7(b), as time goes by, the temperature of the model gradually exceeds the actual temperature. As time goes by, the system gradually reaches a state of thermal equilibrium. In the state of thermal equilibrium, the input and output of heat tend to balance, resulting in the actual temperature tending to stabilize. However, the model may overestimate the accumulation of heat. The difference between the actual and model temperature for all time periods is within a very small range. Table 1 further compares the accuracy test results.

**Table 1:** Control accuracy test results.

Set temperature (°C)	Actual temperature (°C)	Temperature error (°C)
25	25.1	0.1
50	50.2	0.2
75	75.3	0.3
100	100.3	0.3
125	125.1	0.1
150	150.3	0.3
175	175.4	0.4

Table 1 shows the actual achieved temperature and temperature error of the control system at different set temperatures. The smaller the temperature error, the higher the control accuracy of the control system. This indicates that the error between the temperature of set and actual is within 0.5°C, verifying that the research model has high accuracy.

### 3.2 Simulation analysis based on LSTM model

The dataset used for the study is a self-made dataset, with an initial ambient temperature of 26°C and a target set of 120°C. The proportional gain, integral gain, and differential gain are set to 23.5, 120, and 25, respectively. This study selects four different schemes for setting the Initial Learning Rate (ILR), number of hidden units, and maximum iteration times of LSTM. Table 2 shows the parameters of LSTM based on different schemes.

**Table 2:** LSTM parameter list.

Model name	LSTM1	LSTM2	LSTM3	LSTM4
Initial Learning Rate	0.03	0.05	0.08	0.1
Number of Hidden Units	10	20	30	40
Maximum Epochs	60	60	60	60
Optimizer	Adam	Adam	Adam	Adam
Activation Function	tanh	tanh	tanh	tanh
Sequence Length	50	50	50	50
Dropout	0.2	0.2	0.2	0.2

**Fig 8:**
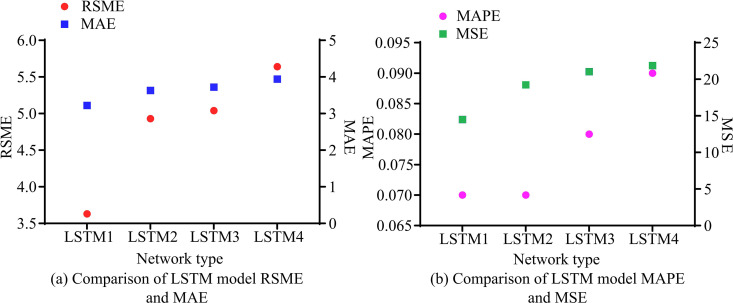
Comparison of prediction accuracy of LSTM model.

Based on the four models in Table 2, this study first compares the prediction accuracy of the four LSTM methods, as exhibited in Fig 8.

**Fig 9:**
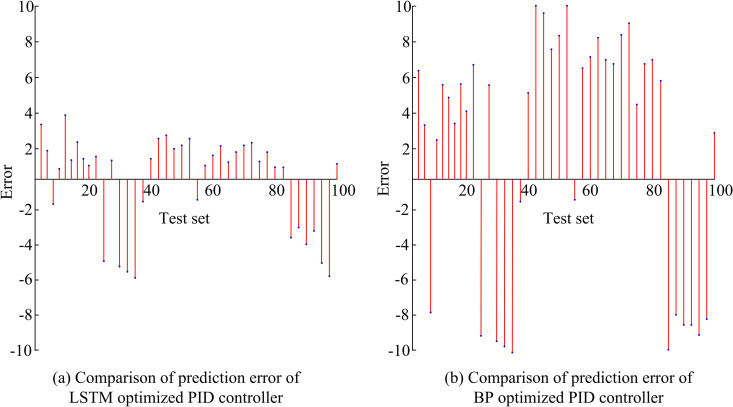
Comparison of PID optimization results between LSTM and BP.

Fig 8(a) shows the comparison of Root Mean Square Error (RMSE) and Mean Absolute Error (MAE) for four LSTM methods. The two error values of LSTM1 are significantly lower than those of other models, with RMSE and MAE of 3.63 and 3.22. Fig 8(b) compares the Mean Absolute Percentage Error (MAPE) and MSE of four types of LSTMs. The MAPE and MSE values of LSTM1 are 0.070 and 14.49, which are significantly lower than those of other LSTM models. In summary, differences in learning rate and hidden layer nodes can lead to biased prediction results. At the same time, when the ILR is 0.03 and the number of hidden nodes is 10, LSTM has the highest prediction accuracy and the smallest error. In the study, the hyperparameters of the model mainly include the initial learning rate, the number of hidden units, the maximum number of iterations, the optimizer, the sequence length and the Dropout rate. Analysis shows that the learning rate determines the step size at which the model parameters are updated in each iteration. A high learning rate may cause the model to fail to converge, while a low learning rate can slow down the training process. The ILR is set to 0.03, which ensures stable convergence of the model and fast training speed. The number of hidden units determines the complexity and memory capacity of the LSTM network. More hidden units can capture more complex features, but they may also lead to overfitting. The 10 hidden units not only avoid the phenomenon that the model cannot capture sufficient features but also improve the expressive ability of the model. This study further explores the prediction error of LSTM-optimized PID (LSTM-PID) based on MATLAB and LSTM1, while introducing BP-optimized PID (BP-PID) for comparison. Therefore, the comparison of prediction errors based on the test set is shown in Fig 9.

**Fig 10:**
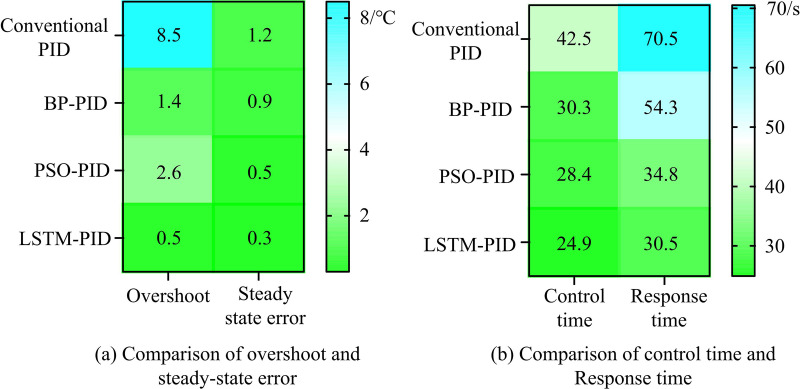
Neural network optimization of PID controller performance parameter comparison.

Fig 9(a) and 9(b) show the error comparison between LSTM-PID and BP-PID controllers. Fig 9(a) shows that the errors are mostly concentrated between −4 and 4. When the number of test sets is around 30 and 90, the errors are the largest, and their values are mostly between −6 and −4. Fig 9(b) shows that overall, the BP-PID controller has significant error fluctuations, with the maximum absolute error value being 10. Overall, the prediction error of the LSTM-PID is smaller than that of the BP-PID. Finally, this study introduces the method described in reference [30] for comparative experiments. Fig 10 shows the performance parameter comparison of PID controller optimized based on neural network.

Fig 10(a) shows the comparison of overshoot and S-SE among four network models. In control systems, overshoot is an important dynamic performance indicator that reflects the stability of system response. The larger the overshoot, the more unstable the system response, which may lead to system oscillation or instability. The smaller the overshoot, the better the stability of the system. S-SE refers to the difference between the system output and the expected output when the system reaches steady state in a control system. This error can be reduced or eliminated by adjusting the proportional gain, integral gain, and differential gain parameters of the PID. This indicates that the overshoot and S-SE of the LSTM-PID controller used are lower than those of the other groups, with values of 0.5 and 0.3, causing an average reduction of 3.67°C and 0.57°C. Fig 10(b) compares the regulation time and response time of four network models. This indicates that the control time and response time of the LSTM-PID controller used are lower, with values of 24.9s and 30.5s, with an average reduction of 8.83s and 22.7s.

### 3.3 Simulation analysis based on WOA-LSTM-PID model

**Fig 11:**
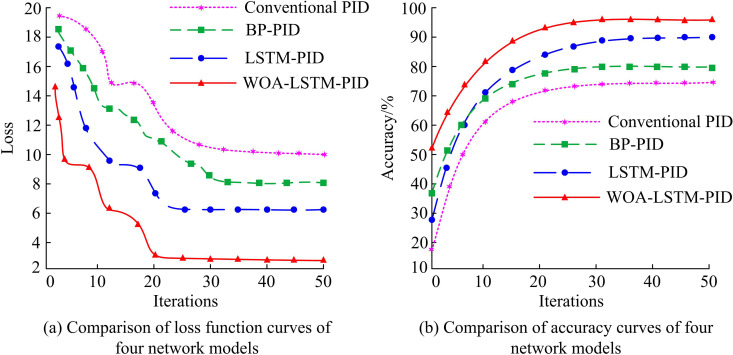
Loss function and accuracy curve comparison.

This study further optimizes LSTM using WOA, with a maximum iteration count of 50 and parameter b = 1. The changes in the algorithm-based loss function and accuracy curve are shown in Fig 11.

**Fig 12:**
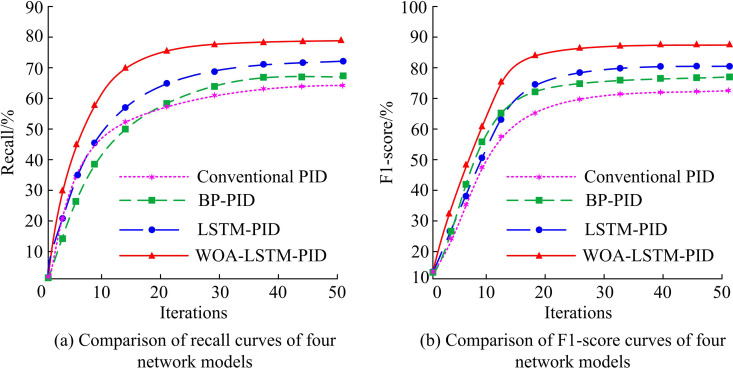
Recall rate and F1-score curve comparison.

In Fig 11(a), as the iteration increases, the loss function gradually decreases. The WOA-LSTM-PID model converges at iteration 20, while its values converge to around 2. Fig 11(b) shows that with the increase of iterations, the accuracy of all four models continues to improve. At the same time, the accuracy of WOA-LSTM-PID after convergence is significantly higher than that of the other groups, and its accuracy eventually converges to 97.9%. The second highest accuracy is LSTM-PID, BP-PID, and Conventional PID. This study further compares the recall rates and F1-score curves of different models, as shown in Fig 12.

**Fig 13:**
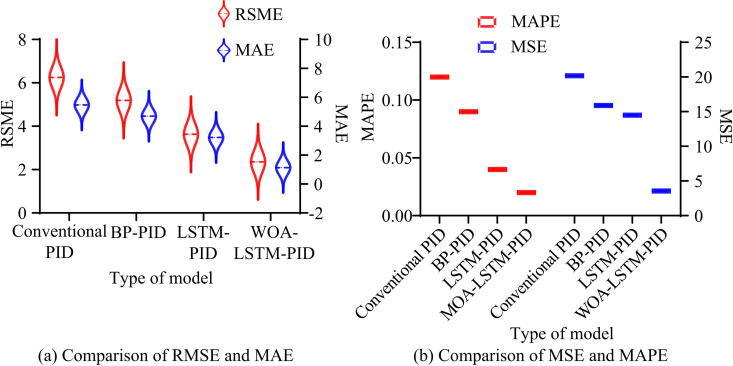
Comparison of error evaluation indexes of four network models.

Fig 12(a) shows that with the growth of iterations, the recall rate gradually grows, and the recall rate of WOA-LSTM-PID converges to 79.5%, which is significantly higher than the other models. Fig 12(b) shows that as the iteration rises, the F1-score gradually rises, and the F1-score of WOA-LSTM-PID eventually converges to 88.9%, with the highest F1-score. The second highest F1-score compared to WOA-LSTM-PID is LSTM-PID, followed by BP-PID and Conventional PID. This study further validates the impact of WOA-LSTM on error evaluation metrics, as shown in Fig 13.

In the comparison of RMSE and MAE in Fig 13(a), after optimizing with WOA, the RMSE and MAE of the LSTM-PID model further decrease, with RMSE decreasing from 3.63 to 2.36 and MAE decreasing from 3.22 to 1.14. In the comparison of Fig 13(b), the MAPE and MSE of WOA-LSTM-PID are the smallest. Compared to LSTM-PID, the error value further decreases after WOA optimization, with MAPE decreasing from 0.04 to 0.02 and MSE decreasing from 14.49 to 3.56. Finally, Table 3 compares the parameters of four network models.

**Table 3:** Performance parameters of four network models.

Model name	Overshoot/°C	S-SE/°C	Control time/s	Response time/s
Conventional PID	8.5	1.2	42.5	70.5
BP-PID	1.4	0.9	30.3	54.3
LSTM-PID	0.5	0.3	24.9	30.5
WOA-LSTM-PID	0.3	0.2	20.2	21.9

In Table 3, all performance parameters of WOA-LSTM-PID are at the minimum numerical level. Compared with LSTM-PID, after WOA optimization, the overshoot of the model decreases from 0.5°C to 0.3°C, and the S-SE decreases from 0.3°C to 0.2°C. The WOA-LSTM-PID used for controlling the temperature of the experimental chamber is more accurate, and the control time and response time have also decreased. The control time has decreased from 24.9s to 20.2 seconds, and the response time has decreased from 30.5s to 21.9s. The overshoot and S-SE of WOA-LSTM-PID are 0.3 and 0.2, which are reduced by an average of 3.2°C and 0.6°C compared to the other groups. In addition, the regulation time and response time of WOA-LSTM-PID are 20.2s and 21.9s, which are an average decrease of 12.4s and 29.9s compared to the other groups. This study compared the computational complexity of four models, namely conventional PID, BP-PID, LSTM-PID and WOA-LSTM-PID, and the results are shown in Table 4.

**Table 4:** Computational complexity indicators of the four models.

Model name	Calculation time/s	Training data volume	Parameter quantity	Memory usage/MB	Iteration
Conventional PID	0.25	/	3	0.5	/
BP-PID	1.87	2000	150	2.3	120
LSTM-PID	3.45	3000	250	4.8	60
WOA-LSTM-PID	2.98	3000	220	3.9	50

In Table 4, the Conventional PID has the shortest calculation time because it is the simplest model, with a value of 0.25 seconds. The calculation time of WOA-LSTM-PID is 2.98 seconds. Although it is shorter than LSTM-PID, it is longer than BP-PID, reflecting the efficiency improvement after WOA optimization. In terms of the amount of training data, WOA-LSTM-PID requires 3,000 samples. However, through WOA optimization, the model can utilize these data more efficiently. In terms of the number of parameters, the Conventional PID has only 3 parameters (proportional gain, integral gain, and differential gain), while the WOA-LSTM-PID has 220 parameters. Through WOA optimization, some redundant parameters are reduced and the model efficiency is improved. In terms of memory usage, the Conventional PID has the smallest memory usage, with a value of 0.5MB. The memory usage of LSTM-PID is 4.8MB because the LSTM network needs to store more status information. The memory usage of WOA-LSTM-PID is 3.9MB, and the memory requirement is reduced through WOA optimization. Finally, in terms of the number of iterations, WOA-LSTM-PID requires 50 iterations. It reduces the number of iterations through WOA optimization and improves the convergence speed. The study further compares the error metrics of the four models Conventional PID, BP-PID, LSTM-PID and WOA-LSTM-PID and the results are shown in Table 5.

**Table 5:** Comparison of error indicators of different models.

Model name	RMSE/°C	MAE/°C	MAPE/%	MSE/°C²
Conventional PID	5.23	3.87	15.4	27.3
BP-PID	2.45	1.89	7.6	6
LSTM-PID	1.23	0.87	3.5	1.5
WOA-LSTM-PID	0.78	0.56	2.3	0.61

In Table 5, the WOA-LSTM-PID model outperforms the other three models in all error indicators. Specifically, the RMSE of the WOA-LSTM-PID model is 0.78°C, which is much lower than that of the other models, indicating that the prediction error of this model is smaller and it can control the temperature more precisely. Its MAE is 0.56°C, which is also the lowest among all models. This means that the average prediction error of the model is the smallest, and the control accuracy is the highest. The MAPE is 2.3%, indicating that the proportion of its error relative to the true value is relatively small, further verifying the high accuracy of the model. Finally, the MSE is 0.61°C², which is also the smallest among all the models. This further demonstrates the stability and accuracy of the model when predicting temperature changes.

## 4. Discussion

The error between the set temperature and the actual temperature of the research model was within 0.5°C, which verified the high accuracy of the thermodynamic model. By comparing four LSTM models with different parameters, it was found that the highest prediction accuracy was achieved when the ILR was 0.03, the hidden units were 10, and the maximum iteration was 60, with an RMSE of 3.63 and a MAE of 3.22. The WOA-LSTM-PID model converged at iteration 20, with the loss function value converging to around 2. The accuracy ultimately converged to 97.9%, the recall rate converged to 79.5%, and the F1 value converged to 88.9%. The results showed that by simulating the social behavior of the whale, the WOA effectively searched the PID parameter space and found the parameter combination that can minimize the control error. Meanwhile, its rapid convergence indicated its high efficiency in optimizing the parameters of complex control systems.

The WOA-LSTM-PID model further decreased in RMSE and MAE compared to LSTM-PID, with RMSE decreasing from 3.63 to 2.36 and MAE decreasing from 3.22 to 1.14. In terms of performance parameters, the WOA-LSTM-PID model had an overshoot of 0.3°C. A lower overshoot meant that the temperature fluctuation of the system was smaller before reaching a steady state. A smaller S-SE of 0.2°C indicated that the system could be maintained at a state close to the set temperature after long-term operation. The control time was 20.2s and the response time was 21.9s. The shorter regulation time and response time indicated that the system could respond quickly to temperature changes. Currently, Hekimoğlu, B et al. optimized PID controllers using a hybrid WOA and Simulated Annealing (SA) algorithm. Similar to the research findings, WOA-SA-PID showed superior performance compared to SA-PID in this study, while significantly improving the transient response of the converter. This study demonstrated that optimizing neural networks with WOA could improve performance and reduce prediction errors [31]. Jassim et al. optimized the parameters of the controller based on the multiverse optimization algorithm. After optimizing the control algorithm, the prediction accuracy of temperature in this study has also been improved, and related parameters such as overshoot and S-SE have been optimized to a certain extent. This study validated the feasibility of optimizing controllers based on control algorithms [32].

In summary, the introduction of WOA-optimized LSTM significantly improves the performance of PID controllers, especially in terms of temperature control accuracy, stability, and response speed. These improvements are crucial for the manufacturing and application of temperature sensitive devices such as OLEDs. The application scope of OLED technology is constantly expanding, from traditional display screens to fields such as flexible displays, transparent displays, and lighting. The WOA-LSTM-PID controller is expected to be widely applied in these fields due to its high precision and fast response characteristics. Meanwhile, in addition to OLED display technology, precise temperature control is equally important for many other high-tech fields, such as semiconductor manufacturing, medical equipment, aerospace, and more. The research results can provide new ideas for temperature control in these fields.

In industrial production, temperature control of OLEDs is crucial for enhancing product quality and extending equipment lifespan. For instance, in the manufacturing process of OLED displays, precise temperature control can ensure the uniformity and stability of light-emitting diodes, thereby enhancing the display effect and service life. Meanwhile, this controller can also be applied in other temperature-sensitive fields, such as semiconductor manufacturing, medical equipment, aerospace, etc. By optimizing PID parameters, the WOA-LSTM-PID controller can effectively cope with temperature changes in complex environments, providing an efficient and reliable solution for industrial automation and intelligent manufacturing.

## 5. Limitations and future work section

However, the WOA-LSTM-PID controller involves multiple hyperparameters (such as the number of hidden units of LSTM, the learning rate, the number of whales of WOA, the number of iterations, etc.), and the adjustment of parameters is relatively complex. Meanwhile, although it performs well in offline optimization and testing, in real-time control systems, the optimization process may not be completed in time, thereby affecting the control effect. To address the limitations of the WOA-LSTM-PID controller, Bayesian optimization, genetic algorithm, or other hyperparameter optimization techniques can be used to automatically adjust the hyperparameters of the model and reduce manual intervention. Meanwhile, real-time optimization algorithms can be developed, such as online learning or incremental learning methods, to enable the model to dynamically adjust parameters during runtime. In addition, the proposed WOA-LSTM-PID controller not only performs well in the experiment but also has significant potential practical application value.

## 6. Conclusion

To improve the temperature control of OLED, this study proposed a WOA-LSTM-PID controller. Compared with traditional PID controllers, WOA-LSTM-PID exhibited significant advantages in temperature control accuracy, stability, and response speed. Its specific manifestation was that WOA-LSTM-PID significantly reduced overshoot and S-SE compared to conventional PID in simulation experiments. Meanwhile, WOA-LSTM performed well in terms of parameters, effectively improving the accuracy of temperature prediction and thus enhancing the performance of PID controllers. In conclusion, the proposed WOA-LSTM-PID controller not only shows excellent performance in the experiments but also has important practical significance for real engineering and industrial applications. By achieving precise and stable temperature control, this controller can directly address the challenges faced by OLED display manufacturing, semiconductor manufacturing, and other temperature-sensitive industries. The improvement of the accuracy and the acceleration of the response time of the WOA-LSTM-PID controller can enhance product quality, reduce production costs, and improve operational efficiency. The research can provide a powerful and adaptable solution that can be easily implemented in industrial environments, contributing to the advancement of these technologies and their applications in various industries.

## Supporting information

S1 File Minimal data set definition. (DOCX)

## Abbreviations:

OLED: Organic Light-Emitting Diode
PID: Proportion Integration Differentiation
WOA: Whale Optimization Algorithm
LSTM: Long Short-Term Memory
RMSE: Root Mean Squared Error
MAE: Mean Absolute Error
MAPE: Mean Absolute Percentage Error
MSE: Mean Squared Error
F1: F1 Score
BP: Back Propagation
SA: Simulated Annealing.
