# Supplementary material for: Improvement analysis of organic light emitting diode temperature control by integrating whale algorithm in PID control system
Source: PLoS One. 2025 Jul 22;20(7):e0327851. doi: 10.1371/journal.pone.0327851 (PMC12282920; doi:10.1371/journal.pone.0327851)
Supplement: S1 File — (DOCX) [file pone.0327851.s001.docx]

**The data in Figure 7(a)**

| Time/min | Actual temperature | Model temperature |
| --- | --- | --- |
| 1 | 19.2 | 19.1 |
| 2 | 40.1 | 39.6 |
| 3 | 59.6 | 59.4 |
| 4 | 80.3 | 80.3 |

**The data in Figure 7(b)**

| Time/min | Actual temperature | Model temperature |
| --- | --- | --- |
| 5 | 92.3 | 92.4 |
| 6 | 123.4 | 123.5 |
| 7 | 146.3 | 146.4 |
| 8 | 156.1 | 156.9 |

**The data in Figure 8(a)**

| Model name | RSME | MAE |
| --- | --- | --- |
| LSTM1 | 3.63 | 3.22 |
| LSTM2 | 4.82 | 3.72 |
| LSTM3 | 5.02 | 3.75 |
| LSTM4 | 5.61 | 3.96 |

**The data in Figure 8(b)**

| Model name | MAPE | MSE |
| --- | --- | --- |
| LSTM1 | 0.070 | 14.49 |
| LSTM2 | 0.073 | 19.46 |
| LSTM3 | 0.082 | 20.27 |
| LSTM4 | 0.089 | 21.96 |

**The data in Figure 9(a)**

| Test set | Error |
| --- | --- |
| 2.24691 | 3.20044 |
| 10.49383 | 3.74529 |
| 4.22222 | 1.62348 |
| 6.74074 | -1.49502 |
| 8.51852 | 0.59579 |
| 14.44444 | 2.13732 |
| 12.64198 | 1.07863 |
| 16.41975 | 1.17165 |
| 25.55556 | 1.04319 |
| 23.20988 | -4.93688 |
| 28.41975 | -5.26024 |
| 30.91358 | -5.54817 |
| 33.4321 | -5.90255 |
| 36.2963 | -1.33555 |
| 38.98765 | 1.14064 |
| 41.30864 | 2.30122 |
| 43.65432 | 2.5227 |
| 45.80247 | 1.72093 |
| 48.49383 | 1.91141 |
| 51.7037 | 2.30122 |
| 56.54321 | 0.75526 |
| 54.22222 | -1.2381 |
| 59.23457 | 1.33555 |
| 62.09877 | 1.91141 |
| 64.61728 | 0.95017 |
| 66.93827 | 1.59247 |
| 69.80247 | 1.94684 |
| 72.32099 | 2.10631 |
| 75.01235 | 0.98117 |
| 77.33333 | 1.59247 |
| 79.85185 | 0.68882 |
| 82.88889 | 0.68882 |
| 85.20988 | -3.52381 |
| 87.7284 | -2.91251 |
| 90.22222 | -3.90919 |
| 92.74074 | -3.13843 |
| 95.60494 | -5.03433 |
| 97.92593 | -5.77409 |
| 99.2716 | 0.85271 |

**The data in Figure 9(b)**

| Test set | Error |
| --- | --- |
| 2.77569 | 6.16643 |
| 4.48792 | 3.02948 |
| 9.07066 | 2.15025 |
| 10.93398 | 5.36231 |
| 12.64621 | 4.61121 |
| 14.78651 | 3.07808 |
| 16.64982 | 5.34022 |
| 20.9304 | 6.47128 |
| 26.09229 | 5.2872 |
| 18.63903 | 3.8822 |
| 39.26138 | 4.88514 |
| 41.67865 | 9.88658 |
| 43.81895 | 9.50661 |
| 46.84054 | 7.42562 |
| 49.25781 | 8.15021 |
| 51.70026 | 9.88658 |
| 56.98804 | 6.31665 |
| 59.4305 | 6.92194 |
| 62.42691 | 8.05301 |
| 64.41612 | 6.82032 |
| 67.43771 | 6.59499 |
| 69.85498 | 8.25183 |
| 72.44851 | 8.87922 |
| 75.01686 | 4.23566 |
| 77.30824 | 6.56849 |
| 80.15357 | 6.79382 |
| 82.873 | 5.56555 |
| 100.77592 | 2.60091 |
| 7.05627 | -7.82171 |
| 23.22178 | -9.20461 |
| 28.38366 | -9.50505 |
| 31.38007 | -9.77899 |
| 34.0995 | -10.13244 |
| 36.54195 | -1.46828 |
| 54.41969 | -1.26505 |
| 85.31545 | -9.95571 |
| 87.8838 | -7.97193 |
| 90.32625 | -8.52421 |
| 93.04568 | -8.52421 |
| 95.61403 | -9.1516 |
| 98.33346 | -8.25028 |

**The data in Figure 10(a)**

| Model name | Overshoot | Steady state error |
| --- | --- | --- |
| Conventional PID | 8.5 | 1.2 |
| BP-PID | 1.4 | 0.9 |
| PSO-PID | 2.6 | 0.5 |
| LSTM-PID | 0.5 | 0.3 |

**The data in Figure 10(b)**

| Model name | Control time | Response time |
| --- | --- | --- |
| Conventional PID | 42.5 | 70.5 |
| BP-PID | 30.3 | 54.3 |
| PSO-PID | 28.4 | 34.8 |
| LSTM-PID | 24.9 | 30.5 |

**The data in Figure 11(a)**

| Conventional PID | | BP-PID | | LSTM-PID | | WOA-LSTM-PID | |
| --- | --- | --- | --- | --- | --- | --- | --- |
| Iterations | Loss | Iterations | Loss | Iterations | Loss | Iterations | Loss |
| 2 | 9.7794 | 2 | 9.3719 | 2 | 8.8258 | 2 | 7.5096 |
| 7 | 9.3719 | 4 | 8.7626 | 4 | 8.2450 | 3 | 6.5721 |
| 10 | 8.6526 | 7 | 8.1676 | 5 | 7.5091 | 4 | 5.2579 |
| 12 | 7.7132 | 9 | 7.5095 | 7 | 6.2889 | 7 | 5.0541 |
| 16 | 7.6991 | 11 | 6.9165 | 11 | 5.3353 | 11 | 3.8009 |
| 19 | 7.0714 | 15 | 6.5721 | 17 | 5.0684 | 17 | 3.2691 |
| 23 | 6.2115 | 21 | 5.8834 | 19 | 4.3022 | 20 | 2.4235 |
| 28 | 5.8202 | 26 | 5.2416 | 25 | 3.8172 | 24 | 2.2992 |
| 32 | 5.6328 | 29 | 4.8340 | 29 | 3.8161 | 30 | 2.2829 |
| 37 | 5.5696 | 32 | 4.6466 | 34 | 3.8003 | 34 | 2.2686 |
| 42 | 5.5391 | 38 | 4.6160 | 39 | 3.7867 | 40 | 2.2645 |
| 46 | 5.5311 | 43 | 4.6629 | 44 | 3.7704 | 44 | 2.2681 |
| 50 | 5.4759 | 50 | 4.6303 | 50 | 3.8009 | 50 | 2.2360 |

**The data in Figure 11(b)**

| Conventional PID | | BP-PID | | LSTM-PID | | WOA-LSTM-PID | |
| --- | --- | --- | --- | --- | --- | --- | --- |
| Iterations | Loss | Iterations | Loss | Iterations | Loss | Iterations | Loss |
| 1 | 16.49771 | 1 | 35.87935 | 1 | 26.87914 | 1 | 51.69655 |
| 3 | 38.01802 | 3 | 50.56039 | 3 | 44.99095 | 3 | 63.86021 |
| 5 | 49.29056 | 5 | 59.67199 | 5 | 60.05071 | 5 | 73.72925 |
| 9 | 60.45171 | 9 | 68.04842 | 9 | 70.56581 | 9 | 81.5933 |
| 14 | 67.6697 | 14 | 73.61715 | 14 | 78.67491 | 14 | 88.56623 |
| 20 | 71.21186 | 20 | 77.67241 | 20 | 83.99929 | 20 | 93.11089 |
| 25 | 72.72675 | 25 | 79.1873 | 25 | 86.65034 | 25 | 97.0045 |
| 30 | 73.61783 | 30 | 80.1898 | 30 | 89.05634 | 30 | 97.51624 |
| 35 | 73.72921 | 35 | 79.69969 | 35 | 89.56873 | 35 | 97.65056 |
| 40 | 73.61786 | 40 | 80.07841 | 40 | 89.81379 | 40 | 97.4055 |
| 45 | 74.10797 | 45 | 79.94474 | 45 | 89.7024 | 45 | 97.9051 |
| 50 | 73.86292 | 50 | 79.43235 | 50 | 89.94745 | 50 | 97.91689 |

**The data in Figure 12(a)**

| Conventional PID | | BP-PID | | LSTM-PID | | WOA-LSTM-PID | |
| --- | --- | --- | --- | --- | --- | --- | --- |
| Iterations | Loss | Iterations | Loss | Iterations | Loss | Iterations | Loss |
| 1 | 0.08321 | 1 | 0.74571 | 1 | 0.05876 | 1 | 0.17706 |
| 3 | 19.72156 | 3 | 13.2622 | 3 | 20.38406 | 3 | 29.28047 |
| 5 | 33.30279 | 5 | 25.897 | 5 | 34.50948 | 5 | 44.32866 |
| 9 | 45.53536 | 9 | 38.27153 | 9 | 46.07955 | 9 | 57.24739 |
| 14 | 52.39695 | 14 | 49.72329 | 14 | 56.58489 | 14 | 69.62192 |
| 20 | 56.98712 | 20 | 58.59605 | 20 | 64.91344 | 20 | 75.39512 |
| 28 | 60.74917 | 28 | 63.96702 | 28 | 69.36165 | 28 | 77.54824 |
| 37 | 63.02059 | 37 | 66.66433 | 37 | 70.97058 | 37 | 79.5344 |
| 43 | 64.10898 | 43 | 67.20853 | 43 | 71.77504 | 43 | 79.5369 |
| 50 | 63.96702 | 50 | 67.32683 | 50 | 72.5795 | 50 | 79.5347 |

**The data in Figure 12(b)**

| Conventional PID | | BP-PID | | LSTM-PID | | WOA-LSTM-PID | |
| --- | --- | --- | --- | --- | --- | --- | --- |
| Iterations | Loss | Iterations | Loss | Iterations | Loss | Iterations | Loss |
| 0 | 2.42405 | 0 | 1.81015 | 0 | 2.55751 | 0 | 1.81015 |
| 3 | 15.3159 | 3 | 17.8782 | 3 | 17.5846 | 3 | 23.9639 |
| 5 | 27.1401 | 5 | 35.1742 | 5 | 30.9303 | 5 | 42.4609 |
| 8 | 40.4856 | 8 | 51.1089 | 8 | 44.5962 | 8 | 55.6731 |
| 12 | 52.1765 | 12 | 61.7320 | 12 | 58.8493 | 12 | 73.1025 |
| 17 | 60.9586 | 17 | 68.6984 | 17 | 72.0348 | 17 | 82.36439 |
| 25 | 65.9759 | 25 | 72.3551 | 25 | 76.8926 | 25 | 85.24705 |
| 32 | 68.2447 | 32 | 73.8765 | 32 | 78.4140 | 32 | 88.4481 |
| 39 | 68.4048 | 39 | 74.0100 | 39 | 78.5742 | 39 | 88.0158 |
| 46 | 69.3123 | 46 | 74.7840 | 46 | 79.0279 | 46 | 88.9083 |
| 50 | 69.0187 | 50 | 74.9175 | 50 | 78.2539 | 50 | 88.9089 |

**The data in Figure 13(a)**

| Model name | RSME | MAE |
| --- | --- | --- |
| Conventional PID | 6.15 | 5.41 |
| BP-PID | 5.23 | 4.95 |
| LSTM-PID | 3.63 | 3.22 |
| WOA-LSTM-PID | 2.36 | 1.14 |

**The data in Figure 13(b)**

| Model name | MAPE | MSE |
| --- | --- | --- |
| Conventional PID | 0.12 | 20.08 |
| BP-PID | 0.09 | 15.11 |
| LSTM-PID | 0.04 | 14.49 |
| WOA-LSTM-PID | 0.02 | 3.56 |
